# How well do plasma Alzheimer’s disease biomarkers reflect the CSF amyloid status?

**DOI:** 10.1136/jnnp-2024-334122

**Published:** 2024-12-18

**Authors:** Jemma Hazan, Emily Abel, Miguel Rosa Grilo, Deborah Alawode, Ines Laranjinha, Amanda J Heslegrave, Kathy Y Liu, Jonathan M Schott, Robert Howard, Henrik Zetterberg, Nick C Fox

**Affiliations:** 1 Division of Psychiatry, UCL, London, UK; 2 UCL, UK Dementia Research Institute, London, UK; 3 UCL Queen Square Institute of Neurology, London, UK; 4 UCL Division of Psychiatry, London, UK; 5 Dementia Research Centre, Institute of Neurology, London, UK; 6 Department of Psychiatry and Neurochemistry, Sahlgrenska Academy, Goteborg, Sweden

**Keywords:** CSF, ALZHEIMER'S DISEASE, DEMENTIA

## Abstract

**Background:**

Can plasma biomarkers as well as cerebrospinal fluid (CSF) perform in the separation of amyloid-beta-positive (Aβ+) vs amyloid-beta-negative (Aβ−) groups across an age range seen in an NHS cognitive disorder clinic?

**Methods:**

As part of the routine diagnostic investigation of 111 clinic patients who had contemporaneous blood and CSF samples taken, patients were categorised into Aβ+ and Aβ− groups based on their CSF in an Aβ42/40 ratio. We then evaluated four single molecule array (Simoa) Quanterix assays, quantifying single plasma analytes and ratios (p-tau217, p-tau217/Aβ42 ratio, p-tau181, p-tau181/Aβ42 ratio and Aβ42/40 ratio) in their ability to distinguish between these groups and the effect of age.

**Results:**

The median (range) age of participants was 66 (55–79) years with 48 females (43.2%). The areas under the curve (AUC), not accounting for age, for the ability to discriminate Aβ+ from Aβ− groups were plasma p-tau217 AUC=0.94, Aβ42/40 AUC=0.78 and p-tau181 AUC=0.77. Combining p-tau217/Aβ42 increased the AUC to 0.97. The difference between the groups was influenced by age with less separation in older individuals: a significant negative interaction term between age and group for plasma p-tau217 concentrations (−0.037, p=0.013) and p-tau217/Aβ42 ratio (−0.007, p=0.008).

**Conclusions:**

There was variable performance of plasma biomarkers to recapitulate the CSF assay. Both p-tau217 and p-tau217/Aβ42 showed excellent promise as surrogates of CSF amyloid status, although with slightly reduced performance in older individuals. There was poorer discriminatory ability for p-tau181 and Aβ42/40. Further research is needed to address potential age-related confounds.

**WHAT IS ALREADY KNOWN ON THIS TOPIC**

- Blood biomarkers have the potential to be used in community memory clinics as a cheap and simple-to-administer test.

**WHAT THIS STUDY ADDS**

- Plasma p-tau217 and p-tau217/Aβ42 showed excellent promise as surrogates of cerebrospinal fluid (CSF) amyloid positivity, although with slightly reduced performance compared to CSF in older individuals.

**HOW THIS STUDY MIGHT AFFECT RESEARCH, PRACTICE OR POLICY**

- These plasma assays exhibit considerable potential as a proxy for CSF, with further work needed to address potential age-related confounds.

## Introduction

 The use of blood-based biomarkers for Alzheimer’s disease (AD) has the potential to enhance the precision of diagnosis in memory clinics, which are predominantly based within mental health services. In these clinics cerebrospinal fluid (CSF) biomarkers are almost never employed, due to limitations in staff skill set and access.^1^,^3^ Blood-based biomarkers offer a more accessible and less-invasive alternative to CSF sampling, without the need for specialist equipment and training.^4^ Several blood biomarkers in the research setting have demonstrated the accuracy in detection of AD pathology.^5^

Blood biomarkers have the potential to be used in community memory clinics as a cheap and simple test.^6 7^ However, careful evaluation is required to assess their performance in a real-world memory clinic population.^7^ CSF biomarkers provide a pre-mortem indication of brain pathology, although their association is not strictly linear, and concentrations can be affected by various pre-analytical and analytical factors.^8^ In contrast, most plasma biomarkers measure CSF proteins, which have crossed the blood-brain barrier to the periphery.^9^ These plasma biomarkers may undergo subsequent peripheral degradation or clearance, and some proteins are also peripherally expressed. There may also be some component of the blood matrix that influences these assays or peripheral biomarker expression.^10^ These factors may introduce discrepancies between CSF and plasma biomarker levels; some of which may be age-related.

Single molecule array (Simoa) ultrasensitive immunoassays provide a highly sensitive and specific method of quantifying AD biomarkers in the plasma.^6 11^ Several AD biomarkers have shown promise and include the amyloid beta (Aβ) species^12 13^ and τ species (both phosphorylated τ (p-tau) and total τ (t-tau)).^6 14 15^

Of particular interest are the plasma assays for p-tau species, which have shown excellent discriminatory ability in research cohorts, distinguishing between cognitive impaired AD, preclinical AD, other neurodegenerative disorders and healthy controls.^16^,^21^

There are relatively few studies assessing the use of these blood biomarkers in clinical practice across a wide age continuum.^22 23^ This is particularly important as there is inconsistency in the literature regarding the association between plasma p-tau concentrations and age.^22 24^ Such an association would have implications for determining reference ranges. We have taken the advantage of a real-world single clinic sample, with heterogeneity in clinical diagnoses, comorbidity and sociodemographic factors. This study aimed to investigate the ability of a blood biomarker panel (plasma p-tau181, p-tau217, p-tau217/Aβ42, p-tau181/Aβ42, Aβ42/40 ratio) in distinguishing between groups in clinical practice who were defined as Aβ+ and Aβ− based on their CSF. We note that historically CSF analyses tried to combine the diagnostic value of both the reductions in Aβ42 and the increases in τ which are features of AD. Therefore, we included ratios of plasma p-tau217/Aβ42 and p-tau181/Aβ42 to assess if there was an added value in detecting CSF-based amyloid positivity as a marker of AD.^25^ We also aimed to investigate the effects of age on this blood biomarker panel in these groups.

## Materials and Methods

### Participants and ethics

This is a cross-sectional study, which included 111 participants investigated for cognitive symptoms at the Cognitive Disorders Clinic at The National Hospital for Neurology and Neurosurgery between August 2013 and January 2023. Patients assessed present with memory difficulties as well as symptoms affecting other cognitive domains. Participants were evaluated with general and neurological examinations. Demographic data including age and sex were collected. Participants underwent a mini-mental state examination, and raw scores are reported in table 1. The diagnostic work up included a standard diagnostic panel of blood tests: brain MRI (unless contraindicated in which case a CT was performed) and formal neuropsychometry. Included participants underwent a lumbar puncture (LP) and donated a blood sample on the same day. The CSF Aβ42/40 ratio was measured with a Lumipulse immunoassay (Fujirebio, Belgium) and used to define an Aβ+ profile, which was classified according to a CSF Aβ42/40 ratio of <0.065 pg/ml.^26^ CSF assays are now getting approval for use in clinical services. One of the CE-marked tests for CSF is the Aβ42/40 ratio, which we used in the main analysis. We also ran a comparative sub-analysis using CSF p-tau181 (online supplemental file 1). Recruited participants included all consecutive cognitive disorder clinic attendees who were being investigated for cognitive complaints and had undergone CSF examination as part of their diagnostic work-up. Participants consented to the use of their samples for research and had paired CSF and blood results available. This sample provided a spread across the age range of 55–79 years.

**Table 1:** Participant characteristics

	Aβ- profile	Aβ+ profile	Total cohort	P value
	(n=51)	(n=60)	(n=111)	
Median age at LP, years (IQR)	66.5 (61.0–72.0)	66.0 (60.0–72.0)	66.0 (60.0–72.0)	0.7310
Age Category (years):				
55–59, N (%)	12 (20.0)	11 (21.6)	23 (20.7)	
60–64, N (%)	13 (21.7)	10 (19.6)	23 (20.7)	
65–69, N (%)	12 (20.0)	13 (25.4)	25 (22.5)	
70–74, N (%)	15 (25.0)	11 (21.6)	26 (23.4)	
75–79, N (%)	8 (13.3)	6 (11.8)	14 (12.6)	
Female, N (%)	20 (33.3)	28 (54.9)	48 (43.2)	0.0223
MMSE, median (IQR)	27 (23–29)	22 (16–26)	25 (20–28)	<0.0001
Aβ+ CSF core biomarkers				
CSF Aβ42/40, median (IQR)	0.11 (0.101–0.112)	0.05 (0.04–0.06)	0.07 (0.05–0.11)	<0.0001
CSF p-tau217, median (IQR; pg/ml)	8.3 (5.6–11.5)	79.1 (53.3–115.7)	20.4 (8.1–74.5)	<0.0001

Data are expressed as median (M) and IQR (age at LP, years, MMSE, AD CSF core biomarkers) or number of participants (n) and percentage (%) (sex).

Aβ+ profile was defined by a CSF Aβ42/40 of<0.065 (Lumipulse G600II, Fujirebio). P values tested the difference between Aβ± core biomarker profile groups and were computed with a Mann–Whitney U test (age at LP, MMSE, AD CSF core biomarkers) or X2 (sex).

Clinical diagnoses of the Aβ− group (n=60: 1 DLB, 11 SCD, 2 MCI, 1 PD dementia, 2 VCID, 4 bvFTD, 4 FTD, 2 FCD, 6 PNFA, 1 meningioma, 1 generalised anxiety disorder, 1 autoimmune disorder, 1 BPAD, 1 chronic fatigue syndrome, 1 depressive disorder, 3 mood-related disorder, 1 unclassifiable dementia, 1 unclear, 5 non-degenerative condition, 1 NPH, 1 PPA, 1 AD dementia, 1 EO AD dementia, 1 CBS, 1 PSP, 5 semantic dementia.

Clinical diagnoses of the Aβ+ group (n=51: 1 SCD, 2 MCI, 4 EO AD dementia, 24 AD dementia, 6 PCA, 8 LPA, 1 FTD, 1 semantic dementia, 2 VCID, 1 PNFA, 1 PPA.

AD, Alzheimer’s disease; Aβ, amyloid beta; BPAD, bipolar affective disorder; bvFTD, behavioural variant of Frontotemporal dementia; CBS, corticobasal syndrome; CDR, Clinical Dementia Rating; CSF, cerebrospinal fluid; EO AD Dementia, early onset AD dementia ; DLB, Dementia with Lewy bodies; FCD, functional cognitive disorder; FTD, Frontotemporal dementia; LPA, logopenic progressive aphasia; MCI, mild cognitive impairment; MMSE, Mini-Mental State Examination; NPH, normal pressure hydrocephalus; PCA, posterior cortical atrophy; PD dementia, Parkinson’s disease dementia; PNFA, progressive non-fluent aphasia; PPA, primary progressive aphasia; PSP, progressive supranuclear palsy; p-tau181, τ phosphorylated at threonine 181; SCD, subjective cognitive decline; VCID, vascular cognitive impairment and dementia.

Participants were excluded if they had any contraindication for LP or did not consent to procedures or lacked matched plasma and CSF measures. Clinical diagnoses of the Aβ- CSF group included dementia with Lewy bodies, subjective cognitive decline, mild cognitive impairment, Parkinson’s disease dementia, vascular cognitive impairment and dementia, behavioural variant frontotemporal dementia, frontotemporal dementia, functional cognitive disorder, progressive non-fluent aphasia, meningioma, generalised anxiety disorder, autoimmune disorder, bipolar affective disorder, chronic fatigue syndrome, depressive disorder, mood-related disorder, unclassifiable dementia, unclear diagnosis, non-degenerative condition, normal pressure hydrocephalus, primary progressive aphasia, AD dementia, early-onset AD dementia, corticobasal syndrome, primary supranuclear palsy and semantic dementia.

Clinical diagnoses of the Aβ+ CSF group included subjective cognitive decline, mild cognitive impairment, early-onset AD dementia and AD dementia. Posterior cortical atrophy, logopenic progressive aphasia, frontotemporal dementia, semantic dementia, vascular cognitive impairment and dementia, progressive non-fluent aphasia and primary progressive aphasia. Characterisation of the cohort, including sample composition, is shown in table 1 in the Results section. The study was approved by a regional ethical committee 12/LO/1504 (Ethics code 120344).

### Plasma and CSF

Non-fasting blood samples were collected in 10 mL ethylenediaminetetraacetic acid-coated tubes. Samples were processed, aliquoted and frozen at −80°C according to the standardised procedures. Plasma analytes (p-tau181, p-tau217, Aβ42 and Aβ40) were measured using commercially available single molecule array (Simoa) assays on the Simoa HD-X platform (Quanterix, Billerica, MA, USA). One of these was a new commercially available Simoa assay for plasma p-tau217 (ALZpath).^21^ CSF p-tau217 was measured with a Lumipulse immunoassay (Fujirebio, Belgium) for this research study, in addition to samples collected as part of routine clinical practice. Collection, processing and storage of CSF samples followed the standardised procedures.^27^ All assays were quantified at the UK Dementia Research Institute at University College London biomarker lab.

### Statistical analysis

Statistical analyses were performed in R v4.1.1. Demographic data, mini-mental state examination (MMSE) scores and plasma biomarker concentrations for the Aβ+ and Aβ− groups were tested for normality using a Shapiro–Wilk test and were compared with a non-parametric Mann–Whitney U test for continuous clinical data variables and Pearson’s X^2^ test for categorical variables. The effect sizes for comparisons were determined by taking the absolute value of the standardised test statistic Z (which represents the magnitude of the difference between groups) and dividing it by the square root of the total number of individuals in the study. Plasma and CSF τ biomarkers levels are presented as median and IQRs. The magnitude of the difference was assessed as percentage increase in plasma and CSF τ biomarker levels for the Aβ+ group compared with the Aβ− group.

We evaluated the precision of plasma biomarkers in distinguishing between Aβ+ and Aβ− groups using receiver-operating characteristic (ROC) analyses by comparing the area under the curve (AUC). Our analysis incorporated logistic regression models to predict CSF Aβ status using single plasma biomarkers or their ratios as predictors. We employed an internal validation method involving five iterations of 10-fold cross-validation and used the pROC package to calculate AUCs and their corresponding 95% CIs. The AUCs of the ROC curves were compared using the DeLong test.

Fitted regression lines and 95% CIs and were generated for plasma and CSF data, stratified by Aβ+ and Aβ− group and plotted by age. Whole group analyses were performed using linear regression models, regressing plasma and CSF biomarker concentrations on age and age and group (Aβ+ vs Aβ−) via an age–group interaction. Linear regression models were performed to examine the association between plasma biomarker concentrations and age separately in the Aβ+ and Aβ− groups.

## Results

### Participants

Participant demographic information and MMSE scores are reported in table 1. The median (IQR) age of all participants was 66.0 (60.0–71.0) years and 43% were female. There was a statistically significant difference in sex (p=0.0223) and mean MMSE scores (p<0.0001) between the Aβ+ and Aβ− groups.

### Plasma biomarker concentrations

The plasma biomarker concentrations are shown in table 2. Plasma concentrations of p-tau181 and p-tau217, and p-tau217/Aβ42 and p-tau181/Aβ42 ratios were significantly greater in the Aβ+ group in comparison to the Aβ− group as indicated by Mann–Whitney U tests (z=5.1, p<0.0001; z=8.1, p<0.0001; z=8.5, p<0.0001, z=6.6, p<0.0001, respectively). There were large effect sizes for p-tau181 (0.5), p-tau217 (0.8), p-tau217/Aβ42 ratio (0.8) and p-tau181/Aβ42 ratio (0.6). A small effect size was found the Aβ42/40 ratio (−0.1), and the ratio was not significantly different between the Aβ+ and Aβ− groups.

**Table 2:** Plasma biomarker concentrations in the amyloid-beta-negative group, Alzheimer’s disease amyloid-beta-positive group and total cohort

Plasma biomarker	Aβ+ CSF profile (Aβ42/40<0.065)						
	Aβ− CSF profile	Aβ+ CSF profile	Total cohort	Z score	P value	Effect size	% increase
	n=60, 54%	n=51, 46%	n=111				
p-tau181, median (IQR; pg/ml)	29.8	46.6	36.0	5.1	<0.0001	0.5	56.4
	(23.6–40.8)	(34.48–61.82)	(28.54–52.16)				
p-tau217, median (IQR; pg/ml)	0.36	1.26	0.7	8.1	<0.0001	0.8	250.0
	(0.26–0.52)	(1.06–1.72)	(0.3–1.2)				
p-tau217/Aβ42 ratio, median (IQR)	0.05	0.24	0.1	8.5	<0.0001	0.8	380.0
	(0.03–0.07)	(0.16–0.32)	(0.04–0.2)				
p-tau181/Aβ42 median (IQR; pg/ml)	3.5	7.5	5.4	6.6	<0.0001	0.6	114.2
	(2.7–5.5)	(5.5–10.5)	(3.4–8.1)				
Aβ42/40 ratio, median (IQR)	0.07	0.06	0.07	−1.3	0.1989	−0.1	−14.3
	(0.06–0.09)	(0.05–0.11)	(0.06–0.09)				

Data are expressed at median (M) and IQR (p-tau181, p-tau217, p-tau217/Aβ42, p-Tau181/Aβ42, Aβ42/40). Z score and p value were calculated using Mann–Whitney U test and effect size calculated by dividing the absolute standardised test statistic Z by the square root of the total number of individuals. Percentage increase (%) calculated as the increase in biomarker concentration in the Aβ+ group compared with the Aβ− group.

Aβ+, amyloid-beta positive; Aβ−, amyloid-beta negative.

The percentage increase, as measured by the increase in biomarker concentration in the Aβ+ group compared with the Aβ− group, is also shown in table 2.

### Discrimination of plasma biomarkers between Aβ+ and Aβ− groups

**Figure 1:**
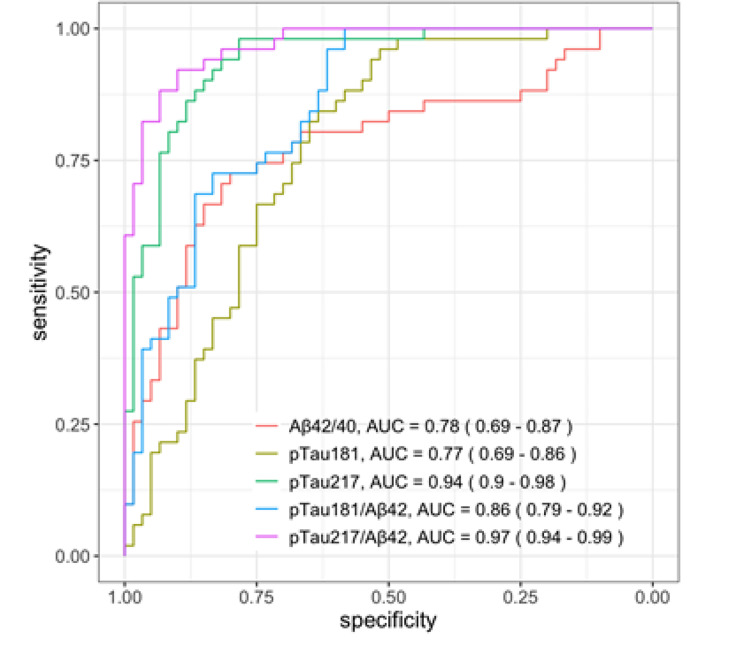
Receiver operating characteristic curve analysis for differentiating amyloid-positive and amyloid-negative groups with plasma biomarker p-tau217, p-tau217/Aβ42, p-tau181, p-tau181/Aβ42 and Aβ42/40.

We examined the discriminatory ability of plasma biomarkers in distinguishing between individuals in the Aβ+ group and those in the Aβ− group using ROC curve analysis (figure 1). The calculated AUCs were from highest to lowest: plasma p-tau217/Aβ42 (0.97, 95% CI 0.94 to 0.99), plasma p-tau217 (0.94, 95% CI 0.90 to 0.98), plasma p-tau181/Aβ42 (0.86, 95% CI 0.79 to 0.92), plasma Aβ42/40 (0.78, 95% CI 0.69 to 0.87) and plasma p-tau181 (0.77, 95% CI 0.69 to 0.86). The DeLong test between AUCs indicated the superior predictive performance of plasma p-tau217 and p-tau217/Aβ42 compared with plasma Aβ42/40 and p-tau181 in distinguishing between Aβ+ and Aβ− groups. Although there were overlapping CIs, there was a significant difference found between plasma p-tau217 and p-tau217/Aβ42 using the DeLong test (online supplemental table 5). The p-tau181/Aβ42 ratio had an inferior performance to p-tau217/Aβ42 ratio and was therefore not included in subsequent analyses.

### Association of plasma biomarker and CSF p-tau217concentrations and age

**Figure 2:**
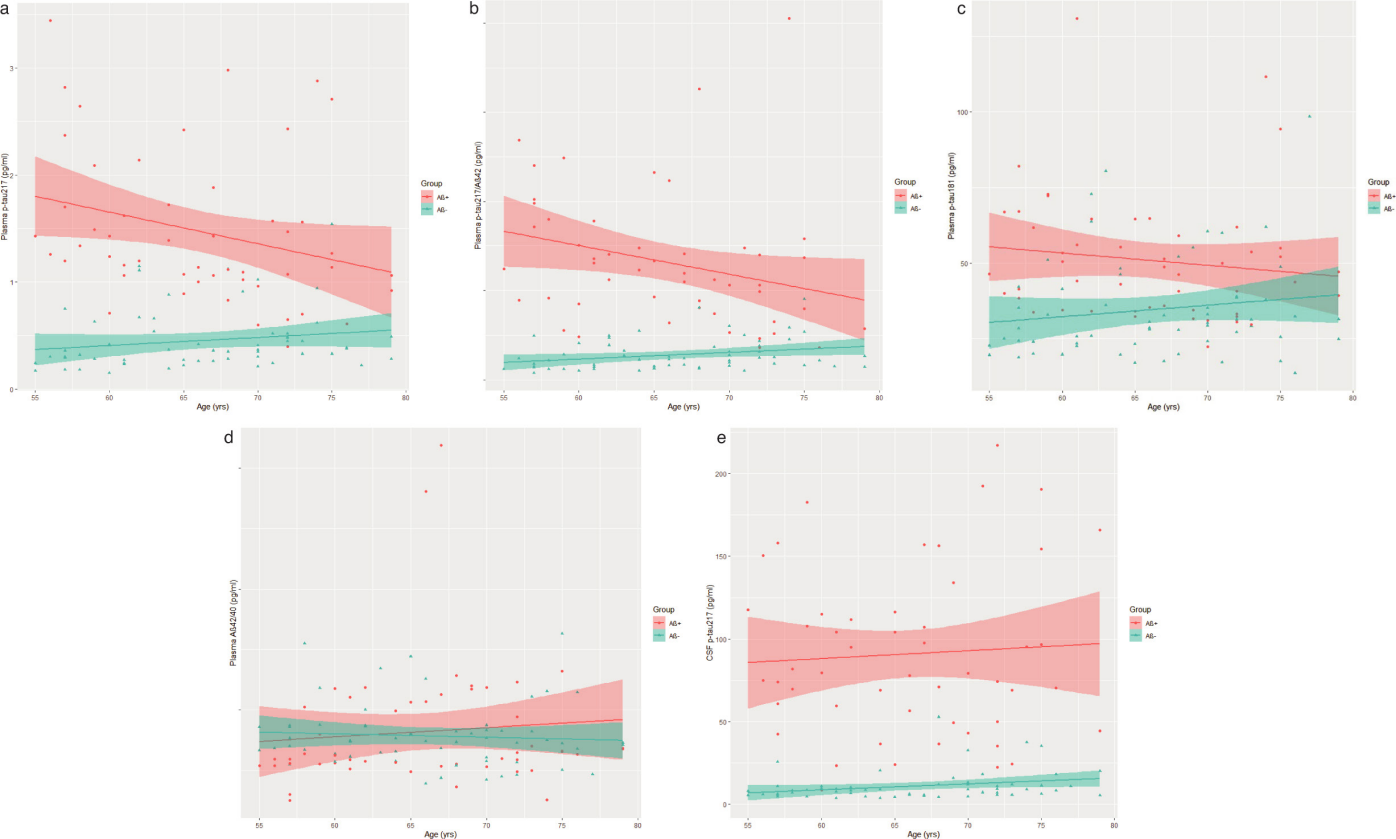
(**a**) Plasma p-tau217 concentrations (pg/ml). Amyloid-positive (Aβ+) participants (red circles) vs amyloid-negative (Aβ−) participants (blue triangles) plotted by age. Data shown with regression lines (line) and 95% CIs (shaded). (**b**) Plasma p-tau217/Aβ42 concentrations (pg/ml). Aβ+ participants (red circles) vs Aβ− participants (blue triangles) plotted by age. Data shown with regression lines (line) and 95% CIs (shaded). (**c**) Plasma p-tau181 concentrations (pg/ml). Aβ+ participants (red circles) vs Aβ− participants (blue triangles) plotted by age. Data shown with regression lines (line) and 95% CIs (shaded). (**d**) Plasma Aβ42/Aβ40 ratio. Aβ+ participants (red circles) vs Aβ− participants (blue triangles) plotted by age. Data shown with regression lines (line) and 95% CIs (shaded). (**e**) CSF p-tau217. Aβ+ participants (red circles) vs Aβ− participants (blue triangles) plotted by age. Data shown with regression lines (line) and 95% CIs (shaded).

We next assessed the association of plasma biomarker concentrations and a CSF biomarker CSF p-tau217 with increasing age. Age-related differences in plasma and CSF p-tau217 biomarker concentrations stratified by group are shown in figure 2. Table 3 shows results from linear regression models of the association between plasma biomarker and CSF p-tau217 concentrations and age, with and without an interaction term (age: group).

**Table 3:** Linear regression analyses examining the association between plasma biomarker p-tau217, p-tau217/Aβ42, p-tau181, Aβ42/40, CSF p-tau217assay concentrations and age and a model incorporating an interaction term age: group

Covariate	Regression coefficients (95% confidence intervals)									
	Plasma p-tau 217 Age Model	Plasma p-tau 217 Age+Age* Group Model	Plasma p-tau217/AB42 Age Model	Plasma p-tau217/AB42 Age+Age*Group Model	Plasma p-tau181 Age Model	Plasma p-tau181 Age+Age*Group Model	Plasma AB42/40 Age Model	Plasma AB42/40 Age+Age*Group Model	CSF p-tau 217 Age Model	CSF p-tau 217 Age+Age* Group Model
Age	−0.012(-0.033, 0.009)	0.007(-0.014, 0.027)	−0.003(-0.007, 0.002)	0.0014(-0.002, 0.005)	−0.018(-0.600, 0.565)	0.381(-0.344, 1.101)	0.0002(-0.001, 0.001)	−0.0003(-0.002, 0.001)	0.220(−1.292, 1.733)	0.362(-0.990, 1.713)
Age*Group†		−0.037(-0.066, −0.008)*		−0.008(-0.0138, −0.002) **		−0.787(-1.860, 0.286)		0.001(-0.001, 0.003)		0.112(-1.889, 2.113)
R2	0.012	0.530	0.014	0.528	<0.0001	0.176	−0.0009	0.010	0.0008	0.575
adjusted R squared	0.003	0.517	0.005	0.482	−0.009	0.152	−0.001	−0.018	−0.008	0.563

*p value map 0 ‘***’ 0.001 ‘**’ 0.01 ‘*’ 0.05 ‘.’ 0.1 ‘ ’ 1.

† non-AD group used as reference.

There were no significant associations between any plasma biomarker or CSF p-tau217 and age for the whole sample. There was, however, a significant negative interaction for the association between plasma p-tau217 concentrations and p-tau217/Aβ42, and age between groups. The Aβ+ group showed a significantly more negative effect of age on p-tau217 concentrations and p-tau217/Aβ42 ratio compared with Aβ− group. For both of these assays, there was a significant negative association with age in the Aβ+ group, as shown in table 4.

**Table 4:** Linear regression analyses examining the association between plasma biomarker p-tau217 and p-tau181 concentrations, p-tau217/Aβ42, Aβ42/40 assay ratios and age in the amyloid-positive and amyloi-negative groups

Disease Group	Regression coefficients (95% CIs)			
	Plasma p-tau 217 Age Model	Plasma p-tau217/AB42 Age Model	Plasma p-tau181 Age Model	Plasma AB42/40 Age Model
Aβ+	−0.030(−0.059, −0.0005)*	−0.006(-0.013, −0.0002)*	0.308(-1.1296, 0.483)	0.0008(−0.002, 0.003)
Aβ-	0.008(-0.004, 0.018)	0.001(-<0.0001, 0.003)*	0.381(-0.276, 1.038)	−0.0003(-0.001, 0.0008)

*p value map 0 ‘***’ 0.001 ‘**’ 0.01 ‘*’ 0.05 ‘.’ 0.1 ‘ ’ 1.

## Discussion

Plasma p-tau217/Aβ42 provided the highest AUC in discriminating between the CSF-based Aβ+ and Aβ− groups. The AUC for plasma p-tau217 was marginally lower than p-tau217/Aβ42, but the 95% CIs overlap between the two. A single assay measure is easier to obtain and calculate alone compared with using a ratio with Aβ42; however, there may be additional benefit from a combination of the biomarkers with little additional cost.

The p-tau217/Aβ42 assay ratio appears promising as a diagnostic measure; comparing its discriminatory ability to p-tau217 needs further study to assess whether it has higher precision and utility in practice. Conversely, both p-tau181 and the Aβ42/40 ratio performed relatively poorly in discriminating between groups.

Both the p-tau217 assay and the p-tau217/Aβ42 ratio assay showed a slightly reduced effectiveness in distinguishing between the groups in older individuals. This differed from CSF p-tau 217 model in which there was not a significant age effect. For both plasma assays, there was a significant negative effect of age in the Aβ+ group. This was an unexpected finding, and there are several explanations which may contribute. This negative effect may reflect altered mechanisms by which p-tau217 moves from the CSF to plasma, possibly reflecting changes in the blood–brain barrier. Younger individuals with cognitive concerns who have Aβ+ CSF are unlikely to have this as an incidental finding. AD pathology is likely to be the cause of their cognitive decline, and as such many individuals have reached a ‘threshold’ for Aβ+ pathology. However, older individuals, such as those with a vascular cognitive impairment or subjective memory concerns, may have Aβ+ CSF without that being the main driver of their presentation. It is important to note that plasma p-tau217 likely reflects both amyloid and τ pathology. These individuals may have diagnoses of mixed disease, or subclinical AD, and as such they will have lower p-tau concentrations correlated with less advanced AD pathology. An alternate explanation is that there is some evidence that older people might have a less aggressive form of AD, with limbic predominance,^28^ and there is evidence that plasma p-tau217 may be less elevated with lower tangle pathology.^20^

Finally, the older participants may have differed in other ways related to differences in co-morbidities, such as renal function or body mass index, which may have affected plasma p-tau concentrations. The effect of age on plasma p-tau concentrations is relatively understudied and requires further evaluation.^20 22 23 29^

A strength of the study is that we used a real-world clinical population comprising of participants who presented to a cognitive disorder service with reported cognitive difficulties and who underwent detailed clinical, cognitive and biomarker assessments. We acknowledge the clinical variability of the participants and that this represents a different approach to the research cohort-based design most commonly used for biomarker studies. We aimed to examine patients with presumed Alzheimer’s pathology based on CSF markers and to assess how AD blood-based biomarkers reflected this. Focusing on Ab+and Ab– groups provided a straightforward and transparent approach for comparing established CSF markers with the blood biomarkers. A limitation of this study is that data for covariates including renal function, body mass index, sociodemographic factors, for example, ethnicity and vascular comorbidities, including prior stroke or cardiovascular disease were unavailable; these covariates would be important to include in further analyses. The participant sample size was another limitation in this study. Another limitation in our study was the relatively small number of individuals with more advanced ages, and the absence of participants over 80 years old. This reflects the ‘real-life’ clinical use of CSF testing. A further limitation was the use of different assay platforms for the measurement of pTau217 in plasma and in CSF (Simoa HD-X and Lumipulse, respectively). Future analyses could aim to quantify this biomarker in different biofluids using the same platform, thus limiting any inter-assay variability, to better understand how plasma biomarker concentrations are related to CSF. Finally, we do not have pathological confirmation of the diagnoses in these cases and so cannot be certain of the final diagnoses or the presence/absence of other comorbidities.

Our findings confirm prior reports that plasma p-tau217 has high accuracy in distinguishing patients with AD and non-AD pathologies as defined using validated CSF biomarkers. Further studies are required to replicate our finding that plasma p-tau217 declines with age; and if so, the mechanisms underpinning this finding and whether it has implications for the use of p-tau217 as a diagnostic test.

These plasma assays exhibit considerable potential as a proxy for CSF.^30 31^ Future directions include assessing the performance of these plasma biomarkers in differentiating AD pathology across different age ranges in large participant cohorts with data on covariates of importance.

## Supplementary material

10.1136/jnnp-2024-334122 online supplemental file 1

10.1136/jnnp-2024-334122 online supplemental file 2

## Data Availability

Data are available upon reasonable request.
