# Supplementary material for: How well do plasma Alzheimer’s disease biomarkers reflect the CSF amyloid status?
Source: J Neurol Neurosurg Psychiatry. 2024 Dec 18;96(6):e334122. doi: 10.1136/jnnp-2024-334122 (PMC12171523; doi:10.1136/jnnp-2024-334122)
Supplement: online supplemental file 1 [file jnnp-96-6-s001.doc]

**Supplementary Data**

Patients were categorized into Aβ+ and Aβ- groups based on their CSF phosphorylated tau181 (p-tau181) levels. CSF p-tau181 was measured with a Lumipulse immunoassay (Fujirebio, Belgium) and used to define an Aβ+ profile, which was classified according to a CSF p-tau181 level of => 57 pg/ml.

**Supplementary Results**

|  | **Aβ- profile** | **Aβ+ profile** | **Total cohort** |  |
| --- | --- | --- | --- | --- |
|  | **(n= 51)** | **(n= 60)** | **(n= 111)** | **p-value** |
| Median age at LP, years (IQR) | 67.5 (60.3-72.0) | 65.0 (60.0-71.0) | 66.0 (60.0-72.0) | 0.411 |
|  |  |  |  |  |
| Age Category (years): |  |  |  |  |
| 55-59, N (%) | 11 (21.6) | 12 (20.0) | 23 (20.7) |  |
| 60-64, N (%) | 13 (25.5) | 10 (16.7) | 23 (20.7) |  |
| 65-69, N (%) | 9 (17.7) | 16 (26.7) | 25 (22.5) |  |
| 70-74, N (%) | 13 (25.5) | 13 (21.7) | 26 (23.4) |  |
| 75-79, N (%) | 5 (9.8) | 9 (15.0) | 14 (12.6) |  |
|  |  |  |  |  |
| Female, N (%) | 18 (35.3) | 30 (50.0) | 48 (43.2) | 0.119 |
| MMSE, median (IQR) | 27 (24-29) | 23 (17-26) | 25 (20-28) | <0.0001 |
|  |  |  |  |  |
| Aβ+ CSF core biomarkers (Lumipulse): |  |  |  |  |
| CSF p-tau181, median (IQR; pg/ml) | 37.3 (29.1-45.4) | 137.8 (83.3-191.0) | 63.3 (41.6-140.3) | <0.0001 |
| CSF p-tau217, median (IQR; pg/ml) | 7.1 (5.6-9.2) | 72.4 (37.2-108.7) | 20.4 (8.1- 74.5) | <0.0001 |

Supplementary Table 1: Participant characteristics

Data are expressed as median (M) and interquartile range (IQR) [age at LP, years, MMSE, AD CSF core biomarkers] or number of participants (n) and percentage (%) [sex].

Aβ+ profile was defined by a CSF p-tau181 of => 57 pg/ml (Lumipulse G600II, Fujirebio). P-values tested the difference between Aβ+/- core biomarkers profile groups and were computed with a Mann-Whitney U test (age at LP, MMSE, AD CSF core biomarkers), or a chi-square (sex).

Clinical diagnoses of the Aβ-group (n =51: 4 bvFTD, 1 chronic fatigue syndrome, 1 Depressive Disorder, 1 LBD, 3 FTD, 2 FCD, 2 MCI, 1 Meningioma, 3 Mood-related disorder, 1 unclassifiable dementia, 3 non-degenerative condition, 1 NPH, 1 PD Dementia, 5 PNFA, 1 PPA, 1 CBS, 1 PSP, 6 semantic dementia, 11 SCD, 1 diagnosis not recorded, 1 VCID

Clinical diagnoses of the Aβ+ group (n = 60: 25 AD dementia, 1 Generalised Anxiety Disorder, 1 Autoimmune Disorder, 1 BPAD, 5 EO AD dementia, 2 FTD, 8 LPA, 2 MCI, 2 Non-degenerative condition, 6 PCA, 2 PNFA, 1 PPA, 1 SCD, 3 VCID

Abbreviations: Aβ, amyloid beta; AD, Alzheimer’s disease; bvFTD, behavioral variant of Frontotemporal dementia; BPAD, bipolar affective disorder; CBS, corticobasal syndrome; CDR, Clinical Dementia Rating; CSF, cerebrospinal fluid; EO AD Dementia, early onset AD dementia ; FTD, Frontotemporal dementia; FCD, functional cognitive disorder; LBD, lewy body dementia; LPA, logopenic progressive aphasia; MCI, mild cognitive impairment; MMSE, Mini-Mental State Examination; ; NPH, normal pressure hydrocephalus; PD dementia; Parkinson’s disease dementia; PCA, posterior cortical atrophy; PNFA, progressive non-fluent aphasia; PPA, primary progressive aphasia; PSP, progressive supranuclear palsy; p-tau181, tau phosphorylated at threonine 181; SCD, subjective cognitive decline; VCID, vascular cognitive impairment and dementia.

|  | **CSF AD CSF Profile (p-tau181 of => 57 pg/ml)** | | | | | | |
| --- | --- | --- | --- | --- | --- | --- | --- |
| **Plasma biomarker** | **Aβ- CSF profile** | **Aβ+ profile** | **Total cohort** | **Z score** | **p-value** | **Effect size** | **% increase** |
|  | **n=51, 46%** | **n=60, 54%** | **n=111** |  |  |  |  |
| p-tau181, median (IQR; pg/ml) | 28.6 | 46.4 | 36.0 | 5.5 | <0.0001 | 0.5 | 62.3 |
|  | (23.4-36.1) | (34.45-59.72) | (28.54-52.16) |  |  |  |  |
| p-tau217, median (IQR; pg/ml) | 0.3 | 1.2 | 0.7 | 7.4 | <0.0001 | 0.7 | 248.5 |
|  | (0.3-0.5) | (0.90-1.61) | (0.3-1.2) |  |  |  |  |
| p-tau217/Aβ42 ratio, median (IQR) | 0.04 | 0.2 | 0.1 | 7.6 | <0.0001 | 0.7 | 376.7 |
|  | (0.03-0.06) | (0.1-0.3) | (0.04-0.2) |  |  |  |  |
| p-tau181/Aβ42 median (IQR; pg/ml) | 3.5 | 7.2 | 5.4 | 6.4 | <0.0001 | 0.6 | 105.3 |
|  | (2.6-5.2) | (5.3-9.8) | (3.4-8.1) |  |  |  |  |
| Aβ42/40 ratio, median (IQR) | 0.08 | 0.06 | 0.07 | -2.0 | 0.05 | -0.2 | 18.03 |
|  | (0.06-0.09) | (0.05-0.1) | (0.06-0.09) |  |  |  |  |

Supplementary Table 2: Plasma biomarker concentrations in the Aβ- group, AD Aβ+ group, and total cohort.

Data are expressed at median (M) and interquartile range (IQR) [p-tau181, p-tau217, p-tau217/Aβ42, Aβ42, Aβ40, Aβ42/40]. Z score and p-value calculated using Mann-Whitney U test and effect size calculated by dividing the absolute standardized test statistic Z by the square root of the total number of individuals. Percentage increase (%) calculated as the increase in biomarker concentration in the Aβ+ group compared to the Aβ- group.


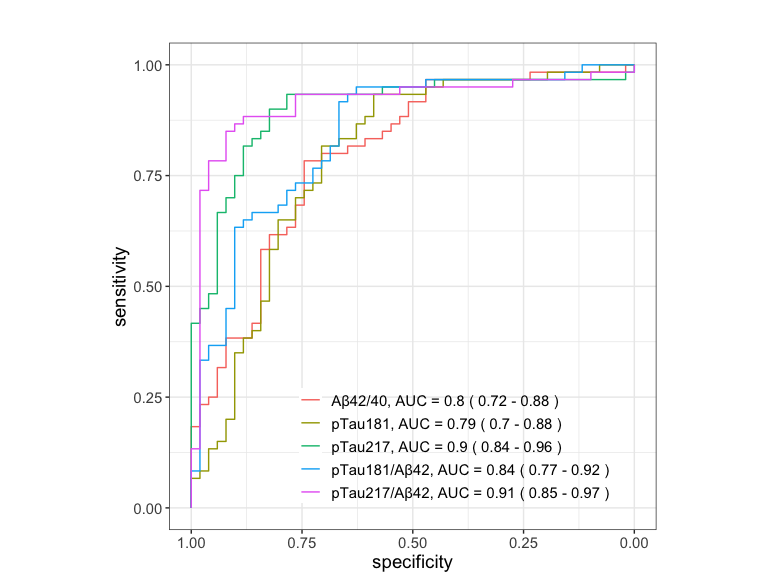


Supplementary Figure 1: ROC curve analysis for differentiating Aβ+ and Aβ- groups with plasma biomarker p-tau217, p-tau217/Aβ42, p-tau181, p-tau181/Aβ42 and Aβ42/40.

|  |  |  |  |  | |  | |  |  |  |  |  | | |
| --- | --- | --- | --- | --- | --- | --- | --- | --- | --- | --- | --- | --- | --- | --- |
|  |  | Regression coefficients (95% confidence intervals) | | | | | | | | | | | |  |
|  | Covariate | Plasma p-tau 217 Age Model | Plasma p-tau 217 Age + Age* Group Model | Plasma p-tau217/AB42 Age Model | | Plasma p-tau217/AB42 Age + Age*Group Model | | Plasma p-tau181 Age Model | Plasma p-tau181 Age + Age*Group Model | Plasma AB42/40 Age Model | Plasma AB42/40 Age + Age*Group Model |  |  |  |
|  |  |  |  |  |  |  |  |  |  |  |  | CSF p-tau 217 Age Model | CSF p-tau 217 Age + Age* Group Model |  |
|  | Age | -0.012 (-0.033, 0.009) | 0.002 (-0.022, 0.026) | -0.002 (-0.006, 0.002) | | 0.0004 (-0.005, 0.005) | | -0.018 (-0.600, 0.565) | -0.094 ( -0.904, 0.714) | 0.0002 (-0.001, 0.001) | -0.0001 (-0.002, 0.002) | 0.220  (-1.292, 1.733) | 0.174 (-1.516, 1.863) |  |
|  | Age*Group¹ |  | -0.034 (-0.066, -0.003)* |  | | -0.007 (-0.014, -0.001) * | |  | -0.062 (-1.135, 1.010) |  | 0.001 (-0.002, 0.003) |  | -0.729 (-2.969, 1.511) |  |
|  | R2 | 0.012 | 0.448 | 0.014 | | 0.421 | | <0.0001 | 0.191 | 0.0009 | 0.0029 | 0.0008 | 0.476 |  |
|  | adjusted R squared | 0.003 | 0.432 | 0.005 | | 0.404 | | -0.009 | 0.169 | -0.008 | -0.025 | -0.008 | 0.462 |  |
|  |  |  |  |  | |  | |  |  |  |  |  | | |
|  | * p value map | 0 ‘***’ 0.001 ‘**’ 0.01 ‘*’ 0.05 ‘.’ 0.1 ‘ ’ 1 | | |  | |  |  |  |  |  |  | | |
|  | ¹non-AD group used as reference | |  |  | |  | |  |  |  |  |  | | |

**Supplementary Table 3: Linear regression analyses examining the association between plasma biomarker p-tau217, p-tau217/ Aβ42, p-tau181, Aβ42/40, CSF p-tau217 assay levels, and age and a model incorporating an interaction term age: group**

|  |  |  |  |  |  |
| --- | --- | --- | --- | --- | --- |
|  | Regression coefficients (95% confidence intervals) | | | |  |
| Disease Group | Plasma p-tau 217 Age Model | Plasma p-tau217/AB42 Age Model | Plasma p-tau181 Age Model | Plasma AB42/40 Age Model |  |
|  |
| Aβ+ | -0.032 ( -0.060, -0.006)* | -0.007 (-0.013, -0.001)* | -0.095 (-0.967, 0.652) | 0.0004 ( -0.002, 0.002) |  |
|  |  |  |  |  |  |
| Aβ- | 0.002 (-0.010, 0.013) | 0.0004 (-0.002, 0.002) | -0.094 (-0.755, 0.565) | -0.0001 (-0.001, 0.001) |  |
|  |  |  |  |  |  |
|  |  |  |  |  |  |
|  |  |  |  |  |  |
| * p value map | 0 ‘***’ 0.001 ‘**’ 0.01 ‘*’ 0.05 ‘.’ 0.1 ‘ ’ 1 | |  |  |  |

**Supplementary Table 4: Linear regression analyses examining the association between plasma biomarker** **p-tau217, p-tau217/ Aβ42, p-tau181, Aβ42/40 assay levels and age in the Aβ+ and Aβ- groups.**


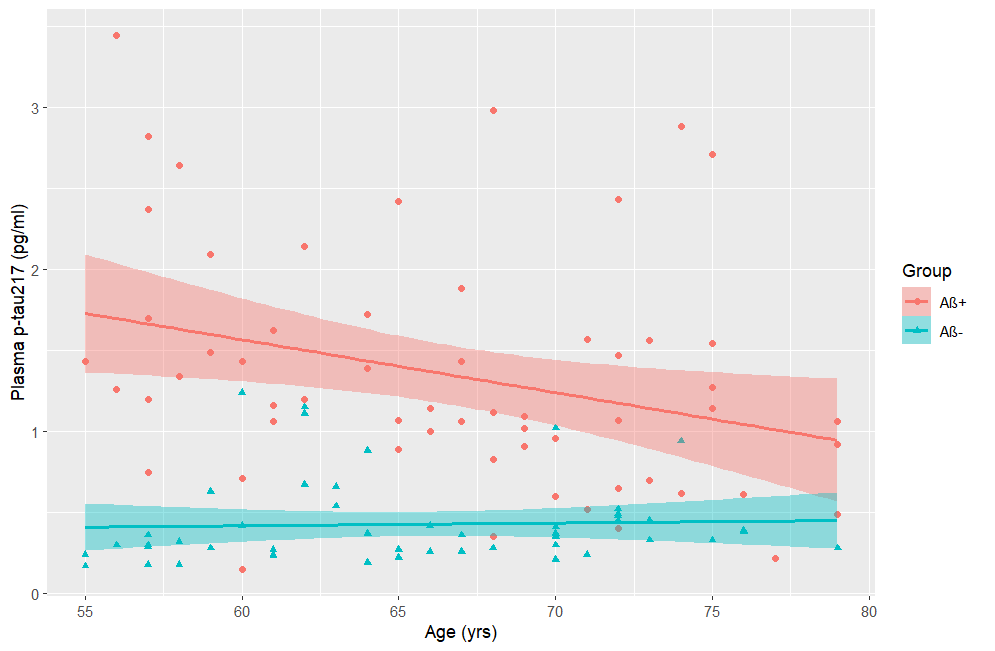


Supplementary Figure 2a: Plasma p-tau217 concentrations (pg/ml). Aβ+ participants (red circles) vs Aβ- participants (blue triangles) plotted by age. Data shown with regression lines (line) and 95% confidence intervals (shaded).

**
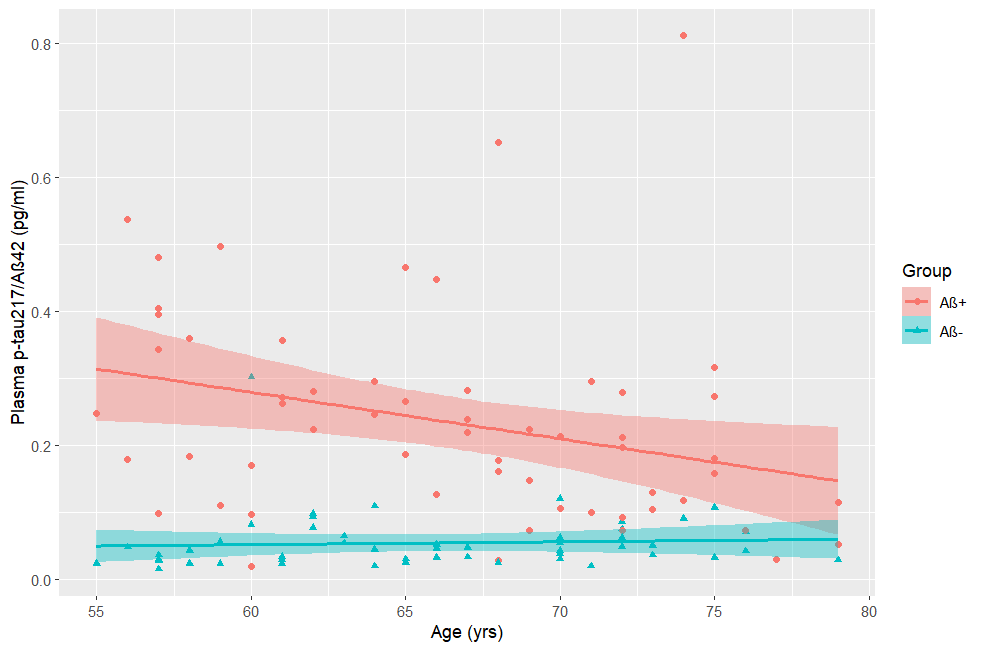
**

Supplementary Figure 2b: Plasma p-tau217/Aβ42 concentrations (pg/ml). Aβ+ participants (red circles) vs Aβ- participants (blue triangles) plotted by age. Data shown with regression lines (line) and 95% confidence intervals (shaded).

**
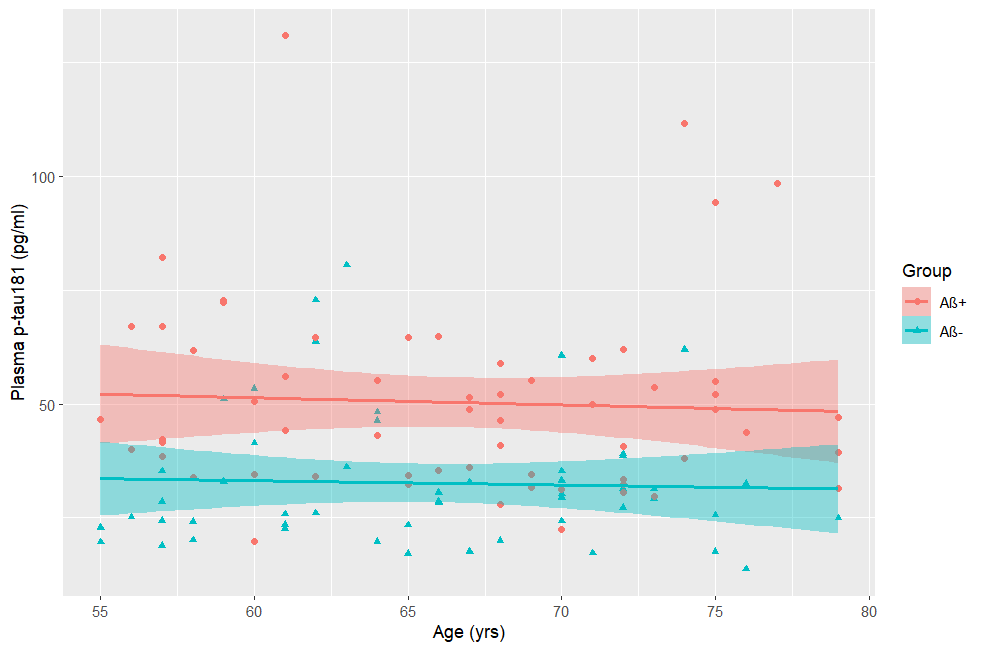
**

Supplementary Figure 2c: Plasma p-tau181 concentrations (pg/ml). Aβ+ participants (red circles) vs Aβ- participants (blue triangles) plotted by age. Data shown with regression lines (line) and 95% confidence intervals (shaded).

Supplementary Figure 2d: Plasma Aβ42/Aβ40 ratio. Aβ+ participants (red circles) vs Aβ- participants (blue triangles) plotted by age. Data shown with regression lines (line) and 95% confidence intervals (shaded).

Supplementary Figure 2e: CSF p-tau217. Aβ+ participants (red circles) vs Aβ- participants (blue triangles) plotted by age. Data shown with regression lines (line) and 95% confidence intervals (shaded).
