# Supplementary figures and images for: How well do plasma Alzheimer’s disease biomarkers reflect the CSF amyloid status?
Source: J Neurol Neurosurg Psychiatry. 2024 Dec 18;96(6):e334122. doi: 10.1136/jnnp-2024-334122 (PMC12171523; doi:10.1136/jnnp-2024-334122)

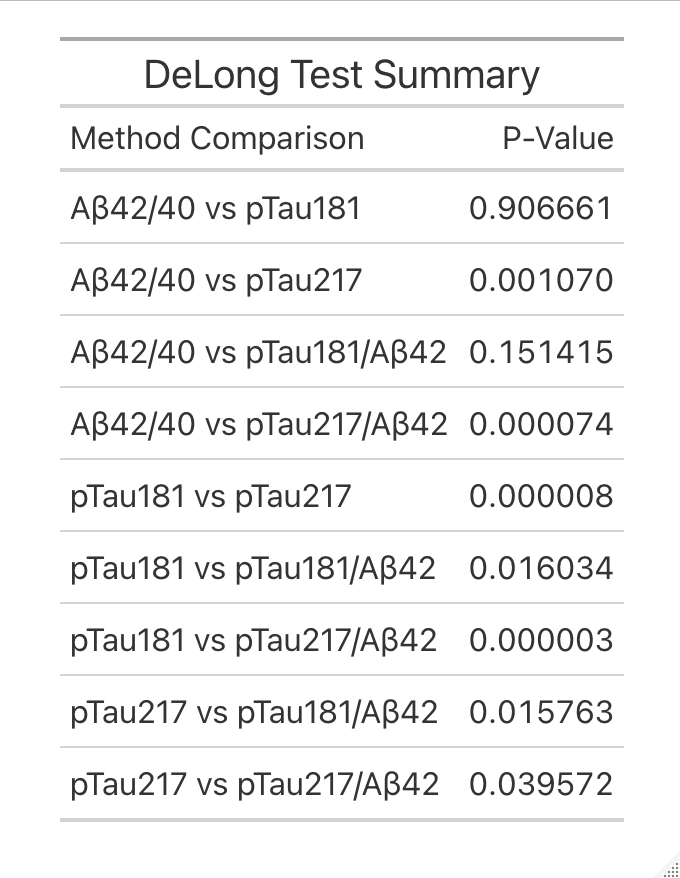


Supplementary Table 5: DeLong Test Summary for comparison of AUCs of the ROC curves

Supplement: online supplemental file 2 [file jnnp-96-6-s002.docx]
